# 16S rRNA sequencing reveals likely beneficial core microbes within faecal samples of the EU protected slug *Geomalacus maculosus*

**DOI:** 10.1038/s41598-018-28720-3

**Published:** 2018-07-10

**Authors:** Inga Reich, Umer Zeeshan Ijaz, Mike Gormally, Cindy J. Smith

**Affiliations:** 1 Applied Ecology Unit, School of Natural Sciences, National University of Ireland Galway, Galway, Ireland; 2 Department of Crop and Soil Science, Oregon State University, Corvallis, OR 97331 USA; 3 School of Engineering, University of Glasgow, Glasgow, Scotland UK

## Abstract

The EU-protected slug *Geomalacus maculosus* Allman occurs only in the West of Ireland and in northern Spain and Portugal. We explored the microbial community found within the faeces of Irish specimens with a view to determining whether a core microbiome existed among geographically isolated slugs which could give insight into the adaptations of *G*. *maculosus* to the available food resources within its habitat. Faecal samples of 30 wild specimens were collected throughout its Irish range and the V3 region of the bacterial 16S rRNA gene was sequenced using Illumina MiSeq. To investigate the influence of diet on the microbial composition, faecal samples were taken and sequenced from six laboratory reared slugs which were raised on two different foods. We found a widely diverse microbiome dominated by *Enterobacteriales* with three core OTUs shared between all specimens. While the reared specimens appeared clearly separated by diet in NMDS plots, no significant difference between the slugs fed on the two different diets was found. Our results indicate that while the majority of the faecal microbiome of *G*. *maculosus* is probably dependent on the microhabitat of the individual slugs, parts of it are likely selected for by the host.

## Introduction

While the study of gut microbial communities is becoming increasingly popular, there is still a dearth of research focusing on those of wild animal populations^1^. This is despite the large influence that factors such as habitat and food availability are likely to have on the gut microbial composition. In fact, it has been shown that captive animals have a distinctly different gut microbiome than those from the wild^2,3^ which is hardly surprising, as a major mode of colonisation of the intestinal tract with microbes is through the environment^4,5^. Hence, the gut microbiome of a species should reflect, at least to an extent, the bacteria which can be found associated with the food or water it ingests in its habitat. Food availability within habitats is, among others, dependent on abiotic factors as well as seasonality and it has been shown that the composition of the gut microbiome of some animals differs between sites and season^6–8^. Additionally, geographical patterns of enteric microbial communities have been discovered in Galapagos iguanas with the microbiota being more distinct the further the islands are separated from each other^9^. While the authors suggest that the dominant drivers of the observed differentiation are host-bacterial interactions and differences in diet, historical and contemporary processes of ecological drift could also be a factor. In *Drosophila*, diet was found to have such a large effect on the gut microbiome that samples clustered by food rather than by host species^10^. Apart from habitat and diet specific microbes the gut harbours a “core microbiome”, members of which have likely co-evolved with their hosts and fulfil important functions including nutrient extraction such as cellulose degradation in termites^11^ or aid with the breakdown of toxins which have been ingested with the diet^12,13^. These bacteria are often specialized gut symbionts and are transmitted vertically from the eggs, through coprophagy or social interactions and it was found that gut communities of social insects were usually more distinctive and consistent than those of non-social invertebrates^4^. There are also indications that some species are deliberately choosing food items which contain byproducts of desirable bacteria to shape their own gut microbiota (e.g. *Drosophila melanogaster*^14^).

Studies of the gut and faecal microbiome of gastropods show that these contain microbes that possess cellulolytic activity^15–17^ and facilitate digestion of lignocellulose by the host^16,17^. This could account for the remarkable efficiency of terrestrial slugs and snails in breaking down plant fibre^18,19^. Cardoso *et al*.^15^ show that a change in diet causes a shift in the gut microbial community of *Achatina fulica*, similar to that observed in humans and other animals, and the authors suggest that the snail gut microbiota might be able to influence the energy balance equation and affect how much energy is extracted from the diet^15^. With only a handful of studies investigating the gut microbiome of slugs, more research is needed to determine the influence of environment and diet on the microbial community of these terrestrial molluscs. The first step is to complete a detailed inventory of the microroganisms associated with the gut of slug. To this end this study focuses on *Geomalacus maculosus* Allman, an EU protected slug species which is found only in the West of Ireland and the North of Iberia. Recent research has shown that the Irish population was probably introduced from Iberia sometime after the last glacial maximum (LGM) and that specimens from different locations within Ireland could not be distinguished using the mitochondrial markers 16 S rRNA and COI^20^. In Ireland *G*. *maculosus* inhabits deciduous and coniferous forests as well as a range of open habitats including blanket bogs and wet grasslands where it feeds on lichens, liverworts, bryophytes and fungi which it grazes from rocks or the bark of trees^21–23^. Hence its gut microbiota might be highly adapted to aid the digestion of non-vascular plants which are staples of its diet.

This study is the first to assess the diversity of bacteria found within faeces of the protected slug *G*. *maculosus*. We employed a two-pronged approach, utilising faecal samples from slugs that were collected from the wild as well as from laboratory hatched specimens to address our aims:

1. To determine whether the slug is a major selector of its microbiome or whether their gut microbes are more reflective of their environment, we collected faecal samples from slugs which were sampled from eleven different sites/seven different habitats. If the former is the case we would expect a substantial ‘core microbiome’ shared by all specimens, if the latter is the case, we hypothesize that the microbial signatures of slugs collected from the same site/habitat will be more similar than those collected from different sites/habitats.
2. To explore the impact of diet on the microbial community composition, we fed laboratory reared slugs from the same egg clutch on two different foods but under the same environmental conditions (e.g. substrate, moisture, temperature). We hypothesize that if diet was the major determinant of the gut microbiome composition, there would be a high degree of separation between the two groups and a high degree of similarity/shared phylotypes within them.

In the light of the protected status of the species, the identification of beneficial microbes could enable predictions about the adaptations of the slug to its habitat and thus help explain its limited distribution. This study also contributes to the further understanding of general invertebrate host-microbe interactions.

## Results

### Microbial diversity

**Figure 1:**
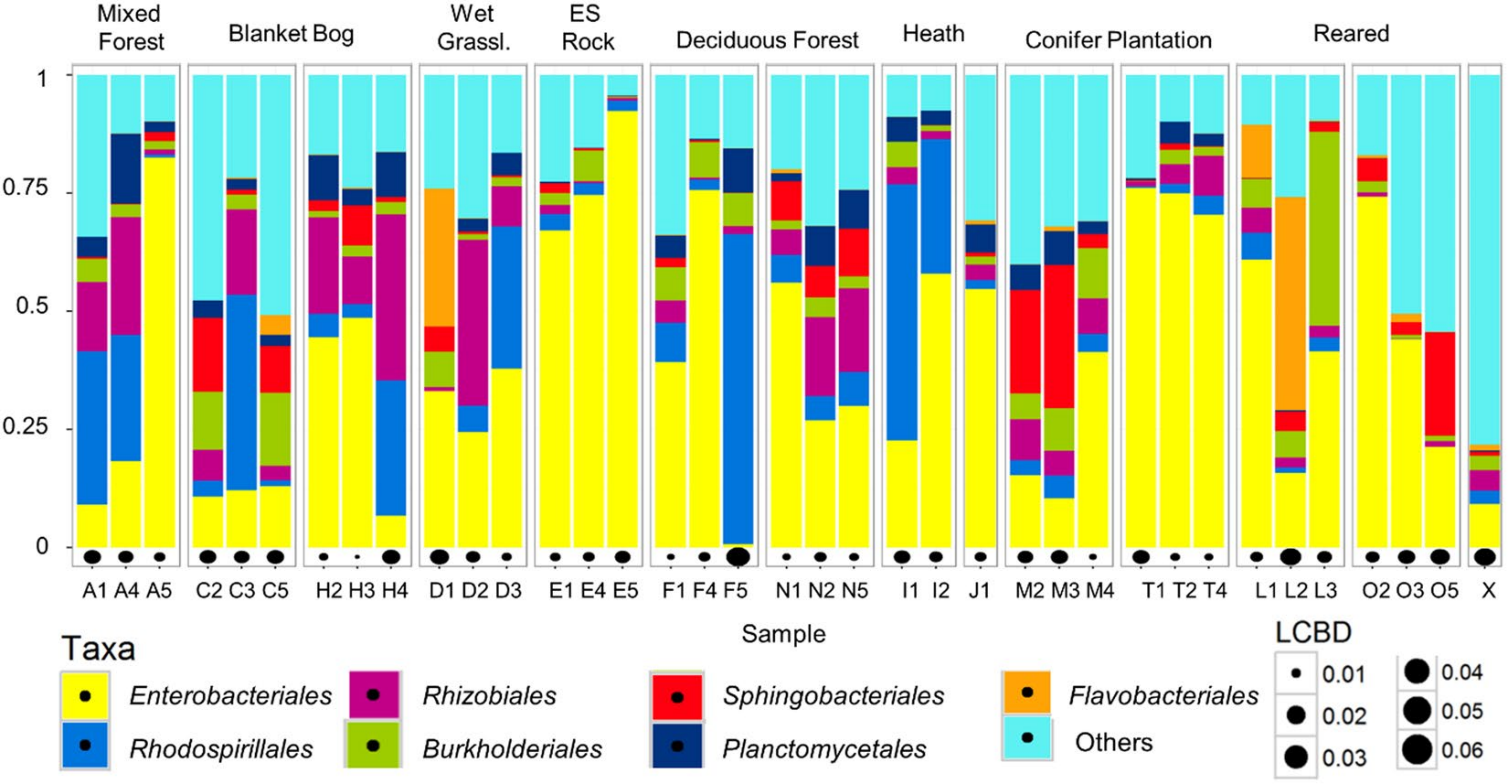
Taxaplot showing all orders with >2.5% abundance within the faecal samples and the LCBD of each sample. They are sorted by sample site (see Fig. 4; L = fed on lichen, O = fed on oats, X = negative control) and grouped by habitat (ES = Exposed Siliceous).

Excluding the negative control, a total of 3,126 Operational Taxonomic Units (OTUs) belonging to 31 phyla and 76 associated classes of bacteria were observed within our samples. The most frequently observed phylum which was dominant in nearly all faecal samples was the *Proteobacteria* (73.1%), followed by *Bacteroidetes* (7.5%). All other phyla apart from *Planctomycetes*, *Acidobacteria*, *Verrucomicrobia*, *Firmicutes* and *Actinobacteria* had an abundance of less than 1%, 6.3% of OTUs were unassigned. The most abundant orders (>2.5%) were *Enterobacteriales* (41.2%), *Rhodospirillales* (10.9%), *Rhizobiales* (7.9%) *Burkholderiales* (5.3%), *Sphingobacteriales* (4.8%), *Planctomycetales* (3.6%) and *Flavobacteriales* (2.7%), however, their abundance between the faecal samples was found to be very variable (Fig. 1). The LCBD values which are shown for each sample (Fig. 1) are a comparative index of uniqueness with large values indicating the samples that have strongly different species compositions compared to the other ones, these include the negative control, most reared specimens and a range of other samples even from within one sample site (Fig. 1). An average of 277 OTUs (±92 standard deviation (SD)) were observed per sample. Many of these were low abundance OTUs: an average of 29.3% (±6.1 SD) were observed as singletons in a sample and an average of 16.6% (±2.7 SD) were observed as doubletons within a sample (Supplementary Table S1).

The negative control contained 234 OTUs, the majority of which belonged to the *Proteobacteria* and *Actinobacteria*, both accounting for more than 40% of the sample (Fig. 1). The most abundant OTU was *Lapillicoccus* (*Actinobacteria*: *Micrococcales*; 20.2%), other abundant OTUs were *Rubrobacter* (*Actinobacteria*: *Rubrobacterales*; 12.4%) and *Acinetobacter* (γ-*Proteobacteria*: *Pseudomonadales*; 11.1%). The dominant OTUs of the negative control were found only in trace abundances in all other samples (≤0.1% of sequences per sample), and 44 of the 234 OTUs of the negative control were not found in any other sample.

### Alpha and Beta Diversity

Significant differences in species richness, Pielou’s Evenness and Shannon’s Diversity Index were found between sites. Samples from Crookhaven and Glengarriff Woods had the highest species richness, which was significantly greater (at P < 0.05) than that at Glanteenassig Forest, Derreen Forest and Cloosh Forest as well as that from the reared specimens. The lowest species richness was recorded from the oat-fed reared specimens (significantly lower than samples collected from all sites except Ballycarbery, Lough Currane, Raferigeen and Derrycunnihy Woods) and from Raferigeen (significantly lower than samples collected from Ballaghbeama Gap and Derreen Forest). Samples from Ballaghbeama Gap had the highest evenness/Shannon Index which was significantly higher than that at Raferigeen, Cloosh Forest, Lough Currane and of the reared specimens. Lough Currane had the lowest evenness/Shannon index which was significantly lower than that at Ballaghbeama Gap and Derreen Forest, (Supplementary Fig. S1).

No clear separation of samples by either sample site or habitat could be observed in the NMDS plots (Supplementary Fig. S2). However, the spread of the samples from the conifer plantation habitat was consistently found to be less than those of the other habitats. No separation of clusters was observed by either the substrate from which the slugs were collected (rock or tree) or by the environment type (forest or open habitat) (Supplementary Fig. S2). While the PERMANOVA found groups to be significantly different when the faecal samples were grouped by sample site (R^2^ = 0.45, *P* = 0.004 (Bray-Curtis); R^2^ = 0.45, *P* = 0.001 (unweighted UniFrac) and R^2^ = 0.42, *P* = 0.03 (weighted UniFrac)), unequal variances could be (partially) responsible for the differences observed between the centroids of the groups which is supported by the absence of clearly separated groups in the NMDS plots (Supplementary Fig. S2).

**Figure 2:**
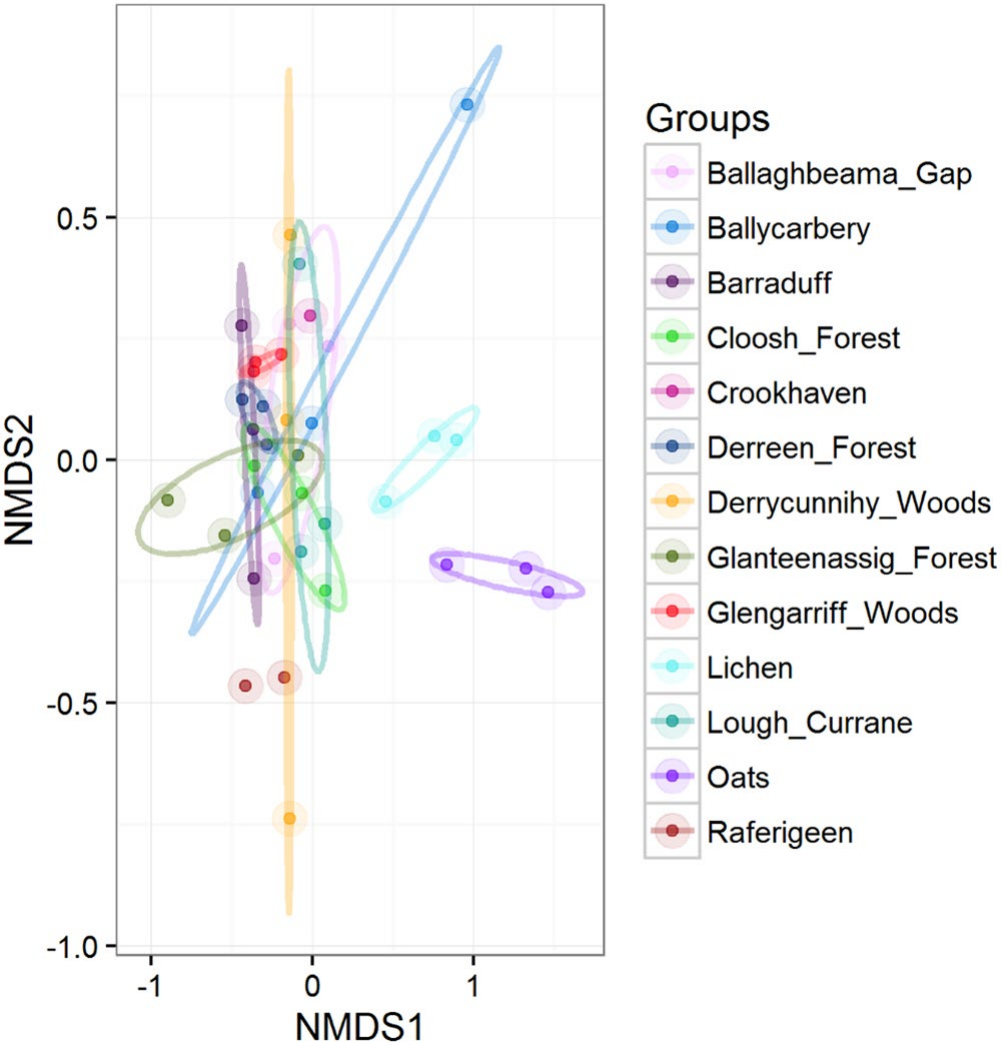
NMDS plot based on Bray-Curtis distances showing the reared and the wild specimens colour coded by sample site (R^2^ = 0.52, *P* = 0.001).

A separation of the reared specimens samples by diet could be observed in the NMDS plots (not shown), however, the sample number was too small (N = 3 for each grouping) that the difference within the groups was not statistically significant despite relatively high R^2^ values (Bray-Curtis: R^2^ = 0.4, *P* = 0.1, unweighted UniFrac R^2^ = 0.36, *P* = 0.1, weighted UniFrac R^2^ = 0.4, *P* = 0.2). When displayed together on an NMDS plot, the reared specimens appeared separated from the wild specimens when using the Bray-Curtis (Fig. 2; R^2^ = 0.52, *P* = 0.001) and unweighted UniFrac (R^2^ = 0.49, *P* = 0.001) distances but not with the weighted UniFrac distances (R^2^ = 0.47, *P* = 0.001; Supplementary Fig. S2).

### Core microbiome

**Figure 3:**
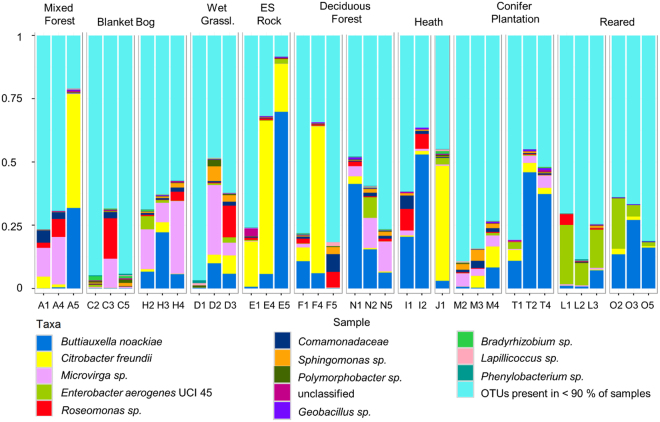
OTUs found in at least 90% of samples (33 of 36) in the order of overall abundance.

Three OTUs (at ≥97% similarity) were found in all faecal samples of the wild and reared slugs, one was an unassigned short read (53 bp) and two belonged to the *Enterobacteriaceae* (*γ-Proteobacteria*: *Enterobacteriales*). One of these was further classified by using blastn and the NCBI database to *Citrobacter freundii* (100% similarity) the other one to *Buttiauxella noackiae* (99% similarity). They accounted for approximately 14% (*B*. *noackiae*) and 9% (*C*. *freundii*) of all sequences respectively and were the most abundant OTUs alongside *Rahnella sp*. (*γ-Proteobacteria*: *Enterobacteriales* 7.2%), *Microvirga sp*. (*γ-Proteobacteria*: *Rhizobiales*; 4.3%) and *Acidiphilium sp*. (*α-Proteobacteria: Rhodospirillales*; 3.4%), which occurred in 89, 94 and 89% of samples respectively. While these five OTUs were dominant in some of the samples, in other samples they accounted for less than 10% of all OTUs. The abundance of the two core OTUs within the samples ranged from <0.1% to 70% with a mean of 13.2% ± 17 SD (*B*. *noackiae*) and 8.3% ±16.3 SD (*C*. *freundii*) respectively. Apart from the three OTUs that were found in all samples, ten further OTUs were observed in at least 90% (=33 specimens) of samples (Fig. 3), with *Microvirga sp*. and an OTU from the family *Comamonadaceae* being significantly more abundant in the wild specimens (*Padj* < 0.001; Supplementary Fig. S3) and *Enterobacter aerogenes* UCI 45 being significantly more abundant in the reared specimens (*Padj* < 0.001; Supplementary Fig. S3). The primary source of the bacterial phylotypes from the NCBI database which were identified as matches to our sequences was soil (Supplementary Fig. S4), followed by sea and sea water, sediment and rhizosphere. In total, 245 biomes (e.g. forest), environmental features (e.g. plantation) or materials (e.g. soil) were assigned to the microbes, indicating that they occur ubiquitously in the environment.

## Discussion

The faecal samples of *G*. *maculosus* were found to harbour a diverse microbial community which differed greatly between individuals. In fact, 40% (1,220) of OTUs were found exclusively in single specimens. Interestingly, the highest number of unique OTUs was from the Crookhaven sample (107 OTUs) where only one slug was sampled indicating that certain OTUs might be associated with specific sites or individual slugs. However, even samples collected from slugs found at the same site were found to differ considerably. This could be explained by the rather sedentary nature of *G*. *maculosus*, which generally does not cover large distances within its habitat^24,25^. As the lichen and bryophyte species differ between habitats and even between trees and rocks within one site, so do the associated microbes which the slugs ingest from the environment through feeding. The observed differences between the faecal bacterial communities of specimens collected from the same site as well as the large amount of low abundance OTUs can hence likely be attributed to micro-structuring within the habitat of the slug.

The lower microbial species richness that we found in the faeces of the reared specimens when compared to the wild slugs has also been observed in a range of other studies^26^. This seems plausible, as they were brought up under more controlled conditions, however, further investigations into biotic and abiotic factors at the sample sites would be necessary to determine why microbial species richness might differ between them.

We found a great overlap of bacterial phylotypes and genera between our study and a range of other studies investigating the gut and/or faecal microbiome of terrestrial slugs^16,17,27^ and snails^15,28–30^, particularly among the *Enterobacteriales* (Supplementary Fig. S5). More than 40% of sequences that were found in the faecal samples of this study belonged to this order, including the two identified core OTUs. These bacteria are part of the gut flora of many animals as well as humans and are also frequently found in water and soil^31^. Genera that were observed in at least four of the eight compared studies (including this work) and could be considered part of a ‘typical’ terrestrial gastropod microbiome include *Aeromonas* (*γ-Proteobacteria*: *Aeromonadales*), *Buttiauxella* (*γ-Proteobacteria: Enterobacteriales*), *Citrobacter* (*γ-Proteobacteria: Enterobacteriales*), *Kluyvera* (*γ-Proteobacteria: Enterobacteriales*) and *Pseudomonas* (*γ-Proteobacteria: Pseudomonadales*) (Supplementary Fig. S5). *Buttiauxella* and *Kluyvera* are frequently isolated from slugs and snails^32^ and the authors even consider molluscs to be the natural source and ecological niche of these bacteria^32^. Additionally, several phylotypes belonging to these genera including *A*. *hydrophilia*, *B*. *agrestis*, *C*. *freundii*, *K*. *intermedia* and *P*. *fluorescens* have been linked with cellulolytic or xylanolytic and pectinolytic activity in the terrestrial slug *Arion ater*^16,17^ and in the silkworm *Bombyx mori*^33^. The likely importance of these bacteria for *G*. *maculosus* is clearly indicated by the presence of *C*. *freundii* and *B*. *noackiae* in all specimens which might serve a key role in the digestion of lichens and bryophytes.

As can be seen by the vast differences in the microbial community composition of our samples (Figs 1 and 3), the environment from which the slugs were collected has most certainly a major impact on the bacteria found in their faeces. However, as discussed above, our sample sites were too complex in structure and too large for a rather sedentary animal to detect site- or even habitat-specific microbial signatures. This is also obvious from the NMDS plots, where there was no clear separation between samples belonging to the same category (Supplementary Fig. S2). Interestingly, conifer plantation was the habitat which was clustering most closely together in the NMDS plots (Supplementary Fig. S2). This could be because conifer plantations in Ireland are predominantly monocultures with a lower species richness of lichens and bryophytes compared to semi-natural woodlands^34,35^ resulting in less diverse food sources for *G*. *maculosus* and hence a less variable microbial community.

Although a separation of the oat and lichen reared slugs was noticeable in the NMDS plots, the sample size was too small to determine whether this was statistically significanant. A total of 702 OTUs were observed in the faeces of the reared slugs, 25 (3.6%) of which were found in all six hatchlings. Out of 386 OTUs observed in the faeces of the oat-fed slugs, 60 (15.5%) were found in all three hatchlings, while 92 out of an observed 516 OTUs (17.8%) were shared by all lichen-fed slugs. This is surprisingly low, considering these slugs were from the same parent and were reared on identical substrate before the samples were taken. Due to the very small size of juvenile faecal pellets, we were forced to wait three weeks before we could collect enough faecal material for DNA extraction. This may have biased the end results, as the DNA extraction was from a composite sample. While the samples were not subjected to changing environmental conditions or shifts in temperatures, a recent study^36^ describes that facultatively aerobic and aerobic bacteria increase while anaerobic bacteria decrease within faecal samples over time, thus affecting the final proportions of taxa. A more precise way of describing the microbial communities found within hatchlings would be the dissection of the juvenile slugs and the examination of the bacteria associated with their gut rather than their faeces.

Three OTUs were shared between all reared and wild specimens and a further 13 were found in at least 90% of samples. This finding indicates that *G*. *maculosus* is a selector of at least part of its microbiome; an assumption which is further supported by the presence of several genera which have the proven ability to aid the digestion in slugs and are common gut bacteria in other terrestrial gastropods^15,16,27–30^. It was mentioned earlier that some *Buttiauxella* and *Kluyvera* strains might in fact be specifically adapted to live inside the gut of molluscs^32^. The ubiquitousness of *Citrobacter* in terrestrial gastropod guts could suggest a similar scenario for certain strains of this genus, however, many of the observed phylotypes (including *Citrobacter*) are rather commonly found in the environment and especially within soil and water, where they can be taken up through feeding. A vertical transfer of some bacteria, which is seen, in particular, among social insects that possess distinctive and consistent gut microbial communities^4^, could also be considered. While *G*. *maculosus* lacks parental care and sociality, a transmission of microbes could occur via the egg. Hatching *G*. *maculosus* slugs do not consume their eggs, even if these are left within the same container for a few days (*pers*. *obs*.), however, they do eat a tiny hole in their egg shell before emerging which might be sufficient for microbial transfer.

In conclusion we showed that the microbial communities found within the faecal samples of *G*. *maculosus* are highly variable even between slugs collected from the same site. We hypothesize that this reflects the significant influence of the microhabitat on the composition of the microbial gut community of *G*. *maculosus*. While diet may influence the gut microbiome of the reared specimens, a larger sample size and a different experimental design are required to further test this hypothesis. To determine the impact of local habitat and feed in shaping the gut microflora of *G*. *maculosus* the microbiome of local food sources should also be considered. The core microbiome consisted of three OTUs which were found within the faecal samples of all wild and reared slugs, at least two of which have likely beneficial functions for their slug host. As similar bacterial phylotypes, many of which have been linked with cellulolytic, xylanolytic or pectinolytic activity, were observed in several other gut microbiota studies on terrestrial molluscs, there is a possibility that these might be selected for by the hosts.

## Methods

### Sampling

#### Wild specimens

**Figure 4:**
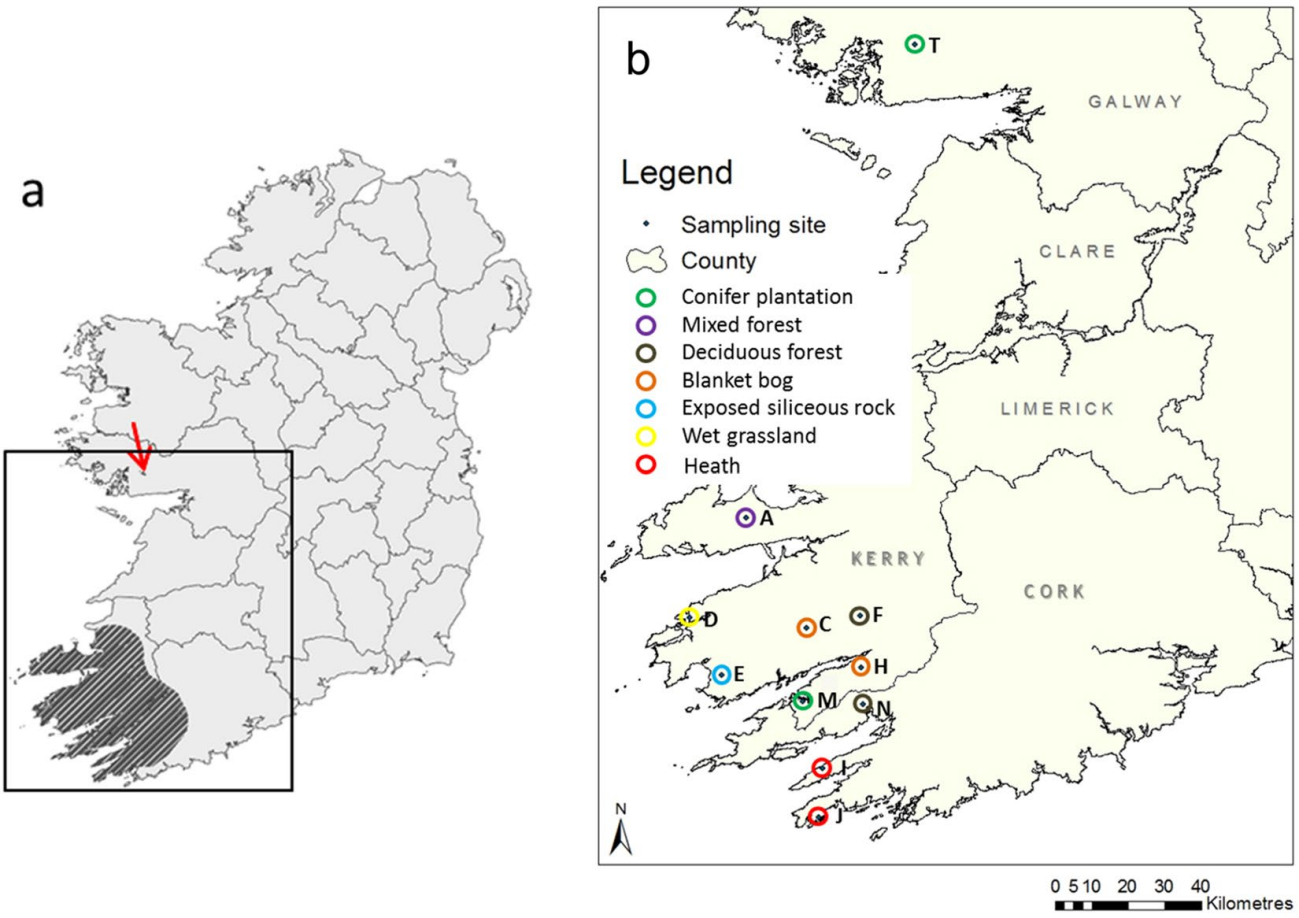
(**a**) The Irish distribution area of *G*. *maculosus* (shaded), the arrow indicates the localised population of the species in County Galway; (**b**) Sites sampled during this study, different habitats are encircled in a different colour. The number of faecal samples used in the following analyses from each site is given in brackets. The maps were generated using ArcGIS 10.2 http://resources.arcgis.com/en/help/install-guides/arcgis-server/10.2/; (**a**) was created by Dr Gesche Kinderman and modified with permission for this publication.

In June and July 2012, 50 *G*. *maculosus* specimens were collected under licence from eleven different locations (between 15 and 200 km apart) within Ireland (Fig. 4). Slugs were sampled from tree trunks or rocks from seven different habitats^37^: blanket bog, heath, exposed siliceous rock, wet grassland, deciduous woodland, mixed woodland and coniferous plantations. They were transferred into sterile petri dishes and observed until they defecated. Freshly collected faeces were transferred into sterile Eppendorf tubes which were initially stored in a mobile freezer compartment at −6 °C before being moved to a −80 °C freezer in the laboratory two days later.

#### Laboratory reared specimens

In June 2014, a clutch of ten eggs was laid by a slug captured two weeks beforehand from the mixed woodland site in Glanteenassig (site A, Fig. 4). The eggs were removed from the parent slug and put into a petri dish containing moist tissue paper and kept at room temperature. After hatching, each slug was transferred into a single petri dish where four slugs were fed with porridge oats, while three slugs were fed with different lichens collected from Cloosh Forest (site T, Fig. 4); three eggs did not hatch. As faecal amounts of the juveniles were small, they were collected over a period of three weeks in the same manner as described above and immediately stored at −80 °C.

### DNA extraction, PCR and sequencing

DNA was extracted from the faecal samples collected from the fifty sampled and six reared slugs using the PowerSoil DNA Isolation Kit (MoBIO, Carlsbad, CA, USA). The V3 region of the 16 S rRNA was amplified with the universal bacterial primers 341 F (5′-CTACGGGAGGCAGCAG-3′) and 518 R (5′-ATTACCGCGGCTGCTGG-3′) using the following conditions: two minutes initial denaturation at 98 °C followed by 30 cycles of 20 seconds at 98 °C, 30 seconds at 58 °C and 30 seconds at 72 °C. The final extension step was for five minutes at 72 °C. One µl of purified DNA was added to a 24 µl PCR mixture containing one unit of Q5 High-Fidelity DNA Polymerase (New England BioLabs, Ipswich, MA, USA) and 0.25 µM of each primer. Each sample was amplified three times and the combined PCR products were run on a 2% agarose gel and subsequently excised and gel extracted using the MinElute Gel Extraction Kit (QIAGEN, Hilden, Germany). As not all 50 samples amplified satisfactorily, the purified PCR products of 36 samples (30 from wild specimens, six from reared specimens) were sent to Research and Testing Laboratory, Texas, USA for sequencing on Illumina MiSeq (sample weight and DNA amount of PCR products are shown in Supplementary Table S2).

The contamination of samples with foreign DNA can pose a problem^38^, especially when working with low microbial biomass samples as in this study. Therefore, a negative control (blank extraction), followed by the same PCR protocol as that of the faecal samples, was also included for sequencing. Additionally, the risk of skewing our results was prevented by using the same extraction kit for all samples^38^.

### Sequence analyses

#### Quality control and pairing

The paired-end reads were filtered and trimmed using Sickle v1.200^39^ by applying a sliding window approach and trimming regions with an average base quality less than 20. A 10 bp length threshold was subsequently applied to discard reads that fall below this length. BayesHammer^40^ from the Spades v2.5.0 assembler was used to error correct the paired-end reads followed by pandaseq v2.4 with a minimum overlap of 50 bp to assemble the forward and reverse reads into a single sequence. This approach was chosen as it has resulted in a reduction of substitution errors by 77–98% with an average of 93.2% for MiSeq datasets in a previous study^41^.

#### Construction of OTU table and phylogenetic tree

After obtaining the consensus sequences from each sample, the UPARSE v7.0.1001 pipeline (https://bitbucket.org/umerijaz/amplimock/src) was used for OTU construction. The reads were barcoded according to sample and pooled together before being dereplicated and sorted by decreasing abundance, singletons were discarded. They were then clustered based on 97% similarity discarding reads that were shorter than 32 bp. Chimeras were filtered using the “Gold” database (http://drive5.com/uchime/ uchime_download.html) that is derived from the ChimeraSlayer reference database in the Broad Microbiome Utilities (http://microbiomeutil.sourceforge.net/). To generate OTU tables for different samples, the original barcoded reads were matched against clean OTUs with 97% similarity. The representative OTUs were then taxonomically classified against the RDP database using the standalone RDP classifier v2.6^42^ with the default–minWords option of 5. OTUs assigned to ‘Chloroplast’, ‘Mitochondria’ and ‘Eukaryota’ were filtered from the OTU table prior to further analysis. To obtain the phylogenetic distances between OTUs, they were multisequence aligned against each other using mafft v7.040^43^. FastTree v2.1.7^44^ was used on these alignments to generate an approximately-maximum-likelihood phylogenetic tree.

#### Statistical analysis

All statistical analyses were performed in R^45^. Alpha diversity (Pielou’s evenness, species richness and Shannon index) was calculated using the package vegan^46^, the *aov* function was used to calculate pairwise ANOVA p-values which were then drawn on top of the alpha diversity figures (see Fig. 1 and Supplementary Fig. S1). Beta diversity was calculated using the packages vegan (Bray-Curtis) and phyloseq.^47^ (weighted and unweighted UniFrac^48^). While Bray-Curtis considers the species abundance count, UniFrac also includes the phylogenetic distance between the branch lengths of OTUs observed in different samples. Weighted UniFrac accounts for the abundance of OTUs and unweighted UniFrac considers their presence or absence. To visualise the similarity of the samples, vegan’s *metaMDS* function was used to produce a non-metric multidimensional scaling (NMDS) plot of community data (OTUs at 3% divergence) based on the Bray-Curtis, weighted and unweighted UniFrac distances. The samples were grouped for different metadata categories (sample site, habitat, substrate and environment type) and standard deviations of the (weighted) averages were drawn as ellipses onto the plot using vegan’s *ordiellipse* function (see Supplementary Fig. S2).

To test whether the centroids and dispersion of the groups differ, vegan’s *adonis* function was used for a Permutational Multivariate Analysis of Variance (PERMANOVA) by partitioning distance matrices among sources of variation (both qualitative and quantitative information). This function fits linear models (e.g., factors, polynomial regression) to distance matrices and uses a permutation test with pseudo-F ratios.

To find OTUs that are significantly different between metadata categories, the function *DESeqDataSetFromMatrix*() from the DESeq2 package^49^ was used, with a significance value cut-off of *P* < 0.001. This function allows negative binomial GLM fitting (as abundance data from metagenomic sequencing is overdispersed) and Wald statistics for abundance data. After correcting for multiple comparisons, it reports OTUs that have log-fold changes between selected metadata categories, in this case between wild and reared specimens (see Supplementary Fig. S3).

We performed Local Contribution to Beta Diversity (LCBD) analysis^50^ by using Hellinger transformation to compute the total sum of squares of the species composition for all samples from which the sample-wise local contributions to beta diversity could be derived as a proportion of the total beta diversity. These values were then plotted as bubbles under stacked bar plots to indicate samples that differ markedly in their species composition (see Fig. 1).

Seqenv^51^ was used to search OTU sequences against the blastn nucleotide database (NCBI), and the textual information on isolation sources of the reference genomes for the OTUs were collated on which we ran a text mining algorithm to identify and parse words associated with the Environmental Ontology (EnvO). The normalized frequencies of EnvO terms for each OTU were then multiplied with the sequence counts from the OTU table to generate a sample-wise EnvO abundance table on which we performed the same differential analysis as we did for OTUs (see Supplementary Fig. S4).

Statistical scripts, workflows, and software used in this manuscript can be found at http://userweb.eng.gla.ac.uk/umer.ijaz#bioinformatics.

### Data availability

Sequencing results are available in the Sequence Read Archive (SRA) database at NCBI under BioProject ID PRJNA386253, accession number SUB2570565.

## Electronic supplementary material

Supplementary Information
