# Supplementary material for: 16S rRNA sequencing reveals likely beneficial core microbes within faecal samples of the EU protected slug Geomalacus maculosus
Source: Sci Rep. 2018 Jul 10;8:10402. doi: 10.1038/s41598-018-28720-3 (PMC6039444; doi:10.1038/s41598-018-28720-3)
Supplement: Supplementary file 1 — Supplementary Information [file 41598_2018_28720_MOESM1_ESM.pdf]

16S rRNA sequencing reveals likely beneficial core microbes within faecal samples of the EU protected slug *Geomalacus maculosus*

Inga Reich, Umer Zeeshan Ijaz, Mike Gormally, Cindy J. Smith

Supplementary Information

Supplementary Table S1. Diversity measures for each sample

Sample site	**Sample ID**	Observed OTUs	Number of singletons	Number of doubletons	Species Richness	Shannon's Diversity Index	Pielou's Evenness
Glanteenassig Forest	**A1**	319	93	58	290.21	3.61	0.63
Glanteenassig Forest	**A4**	269	102	51	241.82	3.36	0.6
Glanteenassig Forest	**A5**	389	124	60	273.16	2.14	0.36
Ballaghbeama Gap	**C2**	444	110	71	357.77	4.4	0.72
Ballaghbeama Gap	**C3**	445	141	90	339.3	3.65	0.6
Ballaghbeama Gap	**C5**	533	103	96	400.65	4.22	0.67
Ballycarbery	**D1**	255	54	35	193.36	3.04	0.55
Ballycarbery	**D2**	364	94	69	298.32	3.5	0.59
Ballycarbery	**D3**	371	116	74	324.62	3.47	0.59
Lough Currane	**E1**	336	62	42	285.55	3.07	0.53
Lough Currane	**E4**	319	117	37	234.89	2.17	0.38
Lough Currane	**E5**	233	87	43	159.09	1.28	0.23
Derrycunnihy Woods	**F1**	575	171	103	421.23	4.14	0.65
Derrycunnihy Woods	**F4**	315	105	59	224.17	2.2	0.38
Derrycunnihy Woods	**F5**	163	50	27	139.69	2.15	0.42
Barraduff	**H2**	445	144	63	344.39	3.46	0.57
Barraduff	**H3**	523	185	100	410.29	3.67	0.59
Barraduff	**H4**	346	85	60	275.54	3.13	0.53
Raferigeen	**I1**	305	85	40	210.72	3	0.52
Raferigeen	**I2**	189	71	25	136.63	2.27	0.43
Crookhaven	**J1**	586	125	98	453.7	3.27	0.51
Derreen Forest	**M2**	422	117	64	331.32	3.97	0.66
Derreen Forest	**M3**	406	139	58	309.16	3.64	0.61
Derreen Forest	**M4**	313	113	44	313	3.73	0.65
Glengarriff Woods	**N1**	348	116	54	284.7	3.02	0.52
Glengarriff Woods	**N2**	560	179	111	417.12	4.02	0.64
Glengarriff Woods	**N5**	573	167	112	454.98	4.02	0.63
Cloosh Forest	**T1**	312	114	50	210.56	2.61	0.45
Cloosh Forest	**T2**	389	154	66	278.86	2.52	0.42
Cloosh Forest	**T4**	294	57	48	248.21	2.82	0.5
Reared - Lichen	**L1**	234	69	54	176.63	2.82	0.52
Reared - Lichen	**L2**	335	73	60	271.69	3.27	0.56
Reared - Lichen	**L3**	260	64	49	180.7	2.79	0.5
Reared - Oats	**O2**	254	77	43	194.52	2.72	0.49
Reared - Oats	**O3**	204	42	26	160.37	2.93	0.55
Reared - Oats	**O5**	159	36	21	119.66	2.65	0.52

Supplementary Table S2 Sample weight [g] and DNA amount [µg/ml] of PCR product

Sample	Weight	DNA amount
A1	0.14	17.7
A4	0.08	17.3
A5	0.06	14.6
C2	0.06	14
C3	0.07	21.5
C5	0.07	25.6
D1	0.07	25.2
D2	0.1	17
D3	0.08	26.9
E1	0.09	36.8
E4	0.15	16
E5	0.08	20
F1	0.16	25.2
F4	0.12	23.5
F5	0.12	18.9
H2	0.04	15.5
H3	0.03	22.8
H4	0.02	11.2
I1	0.05	12.7
I2	0.05	27.9
J1	0.02	13.1
M2	0.18	32.9
M3	0.17	21.8
M4	0.13	18.3
N1	0.08	25.1
N2	0.02	33
N5	0.09	15.5
T1	0.08	17.9
T2	0.05	25.8
T4	0.12	12.9
L1	n/a	15.8
L2	n/a	30.1
L3	n/a	15.1
O2	n/a	23.2
O3	n/a	26.3
O5	n/a	19.1

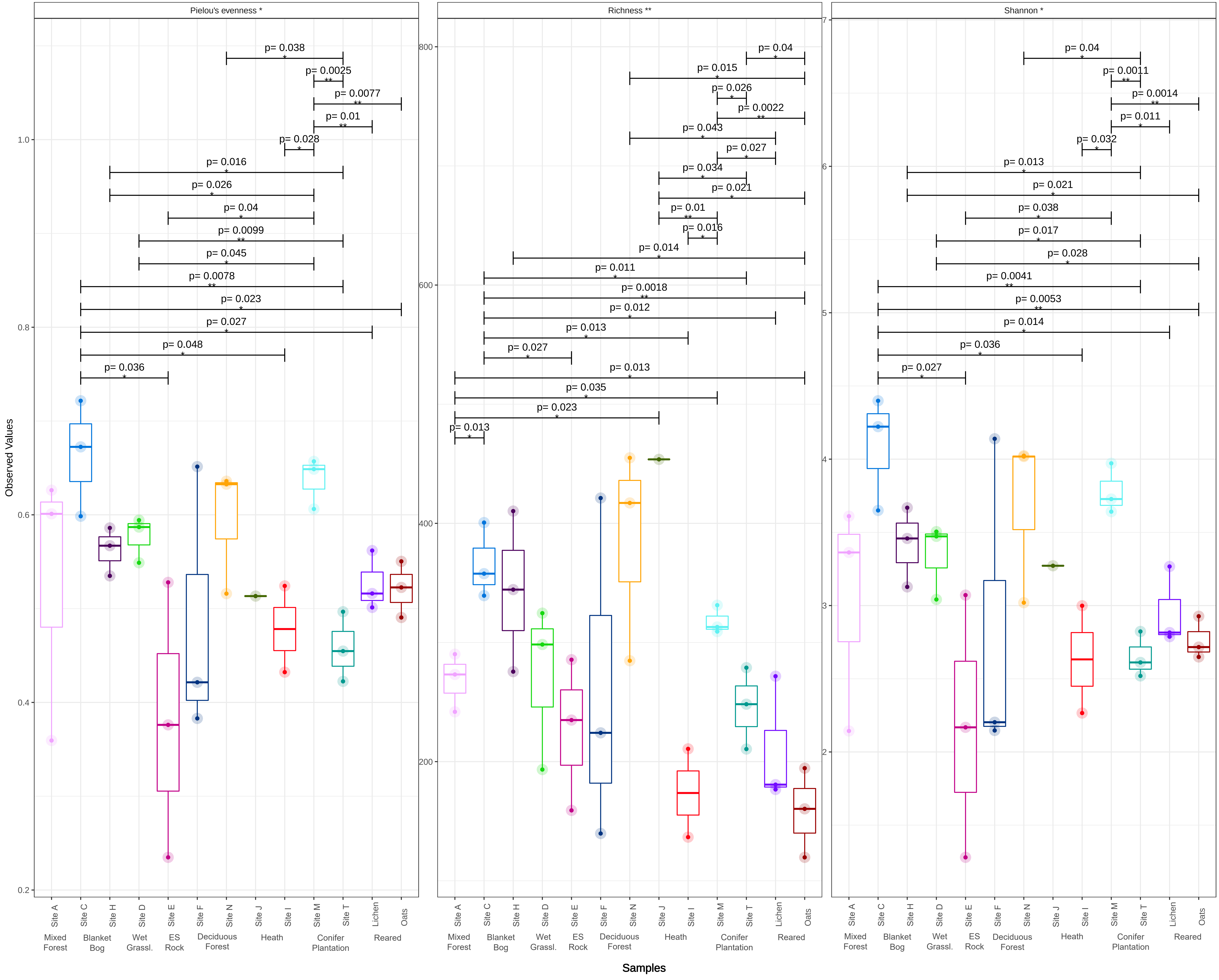

Supplementary Figure S1. Pielou's evenness, richness and Shannon's Diversity Index for each sample (dots) grouped by site and arranged by habitat (ES = Exposed Siliceous). The thick horizontal line is the median sample value while the box shows the interquartile range. Statistically significant differences between sites at $p < 0.05$ are shown above the plot.

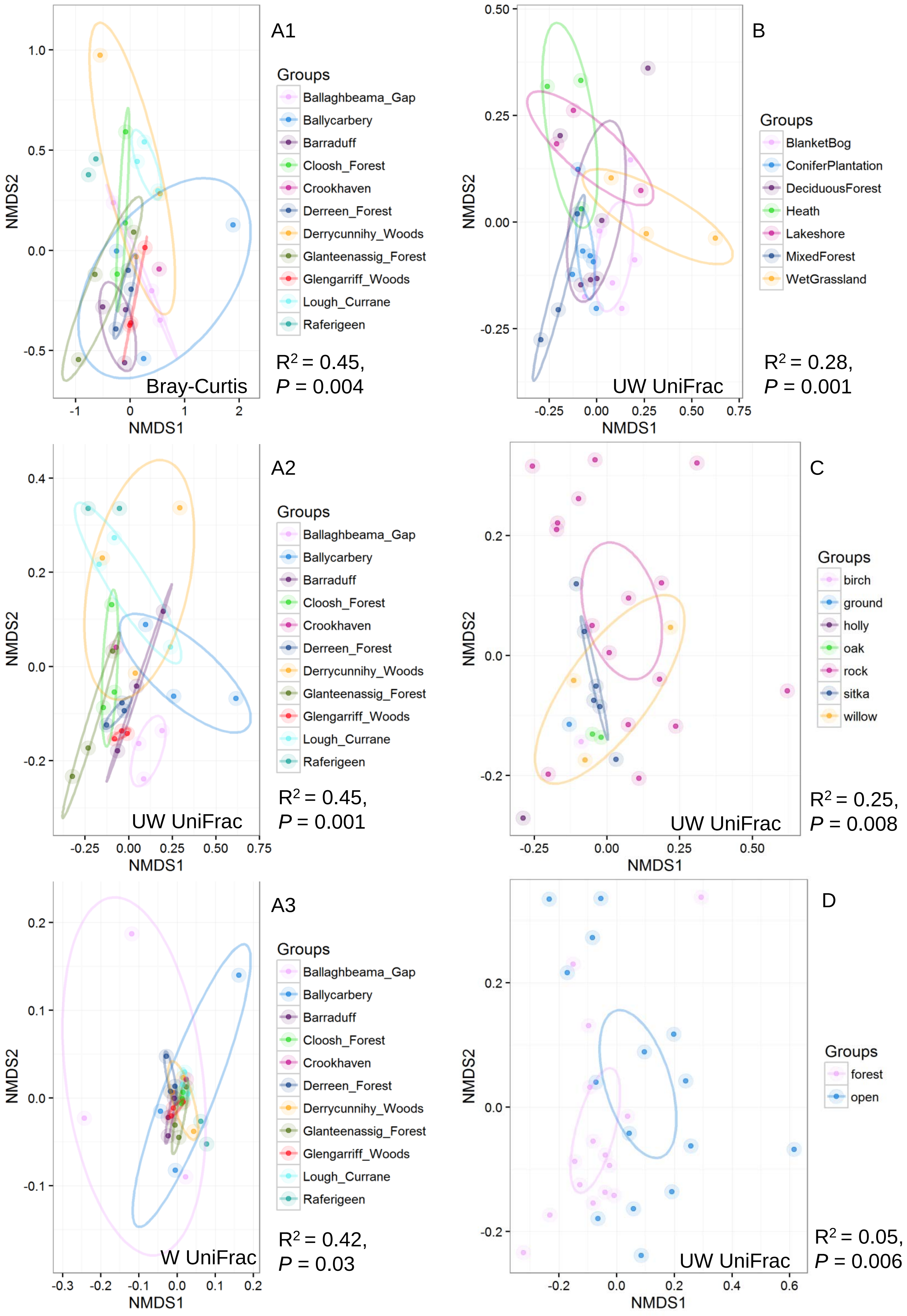
A1
Groups
Ballaghbeama_Gap
Ballycarbery
Barraduff
Cloosh_Forest
Crookhaven
Derreen_Forest
Derrycunnihy_Woods
Glanteenassig_Forest
Glengarriff_Woods
Lough_Currane
Raferigeen
R2 = 0.45,
P = 0.004
Bray-Curtis
NMDS1
NMDS2
B
Groups
BlanketBog
ConiferPlantation
DeciduousForest
Heath
Lakeshore
MixedForest
WetGrassland
R2 = 0.28,
P = 0.001
UW UniFrac
A2
R2 = 0.45,
P = 0.001
UW UniFrac
C
Groups
birch
ground
holly
oak
rock
sitka
willow
R2 = 0.25,
P = 0.008
UW UniFrac
A3
R2 = 0.42,
P = 0.03
W UniFrac
D
Groups
forest
open
R2 = 0.05,
P = 0.006
UW UniFrac

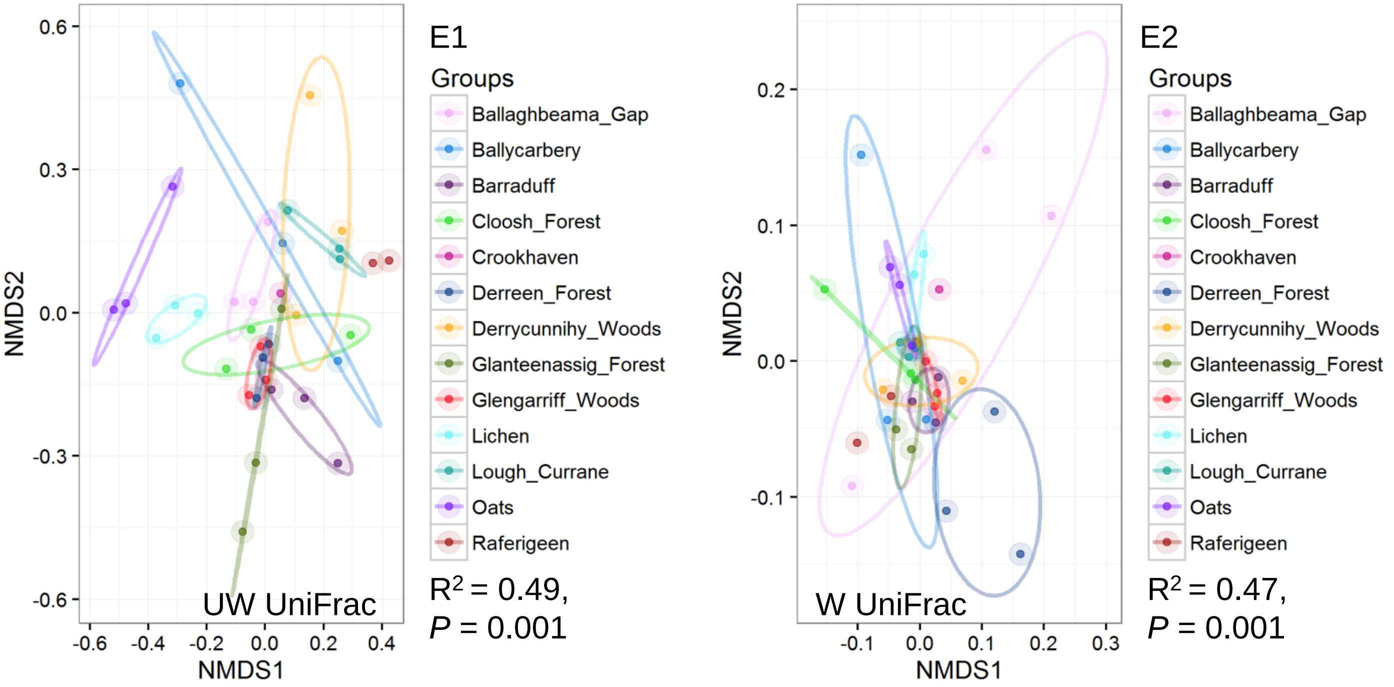

Supplementary Figure S2. NMDS plots based on Bray-Curtis, unweighted (UW) and weighted (W) UniFrac distances and colour coded by sample site (A, E), habitat (B), substrate (C) or environment type (D). E1 and E2 also include the reared specimens. Only plots with $P < 0.05$ are shown.

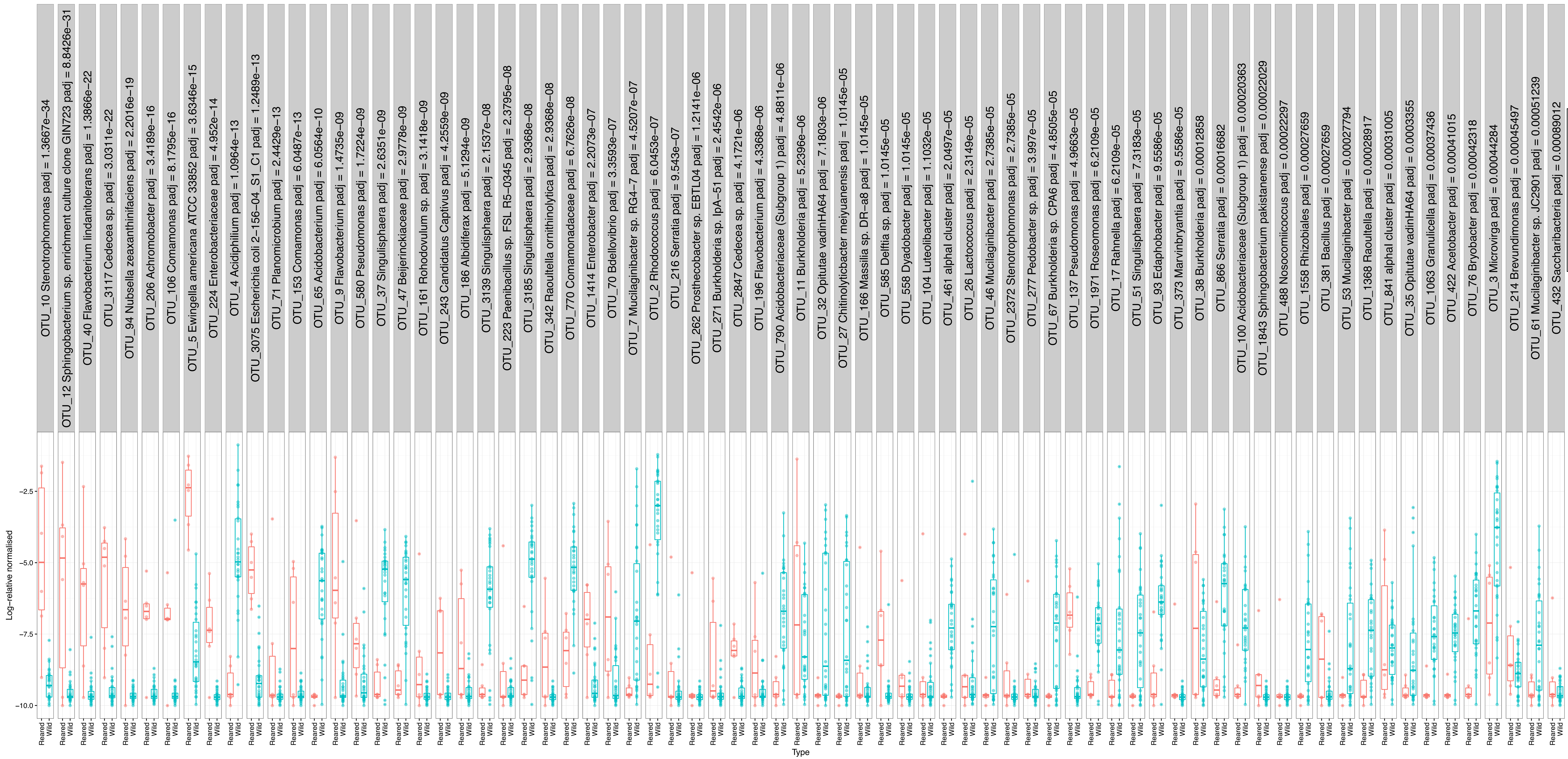

Supplementary Figure S3. OTUs with log-fold changes between reared and wild specimens (OTUs without any classification not shown).

Supplementary Figure S4. The most common isolation sources of the bacteria from the NCBI website which were identified as matches to the sequences in this study

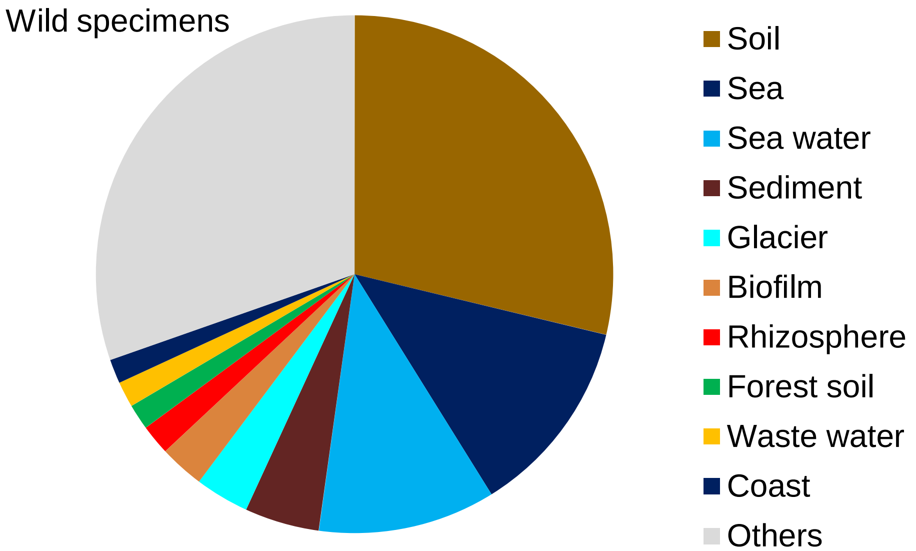

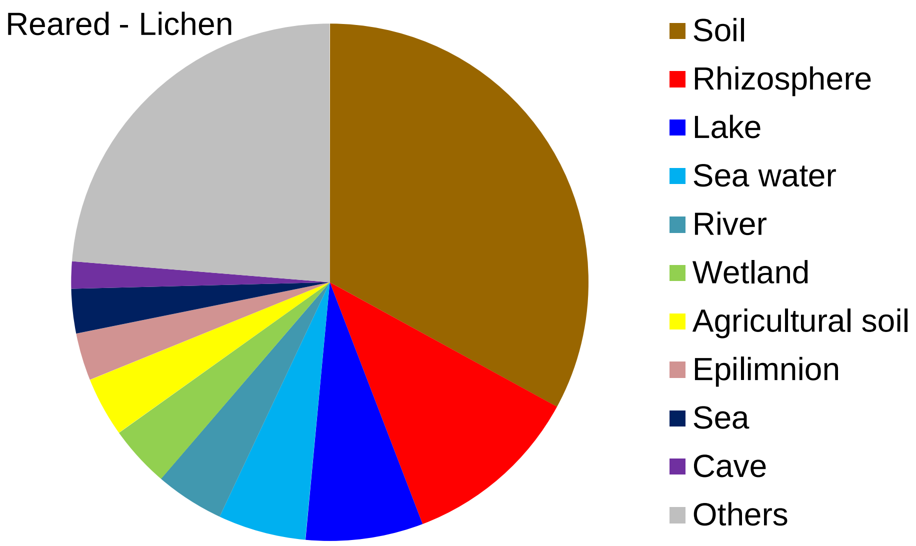

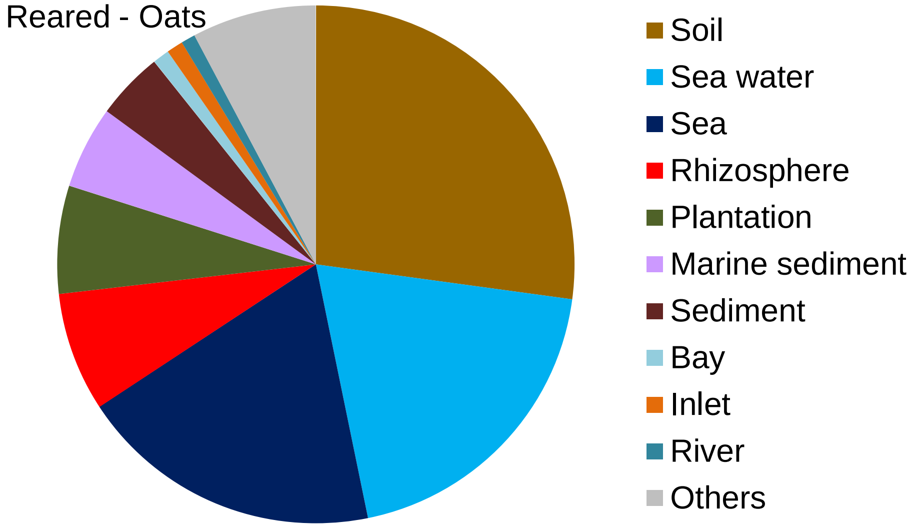

Supplementary Figure S5. Bacteria isolated from guts, crops and faeces from other studies investigating terrestrial snails and slugs (only genera mentioned in more than 1 study are included)

Class	Order	Family	Genus	Cardoso et al. 2012	Charrier et al. 1980	Joynson et al. 2014	Joynson et al. 2017	Rossing & Hietpas 2007	Stalder et al. 2014	Wilkinson 2010	This study	Observed studies
			**Organism(s)**	*Achatina fulica* (land snail)	*Helix pomatia*, *Cornu aspersum* (land snails)	*Arion ater* (terrestrial slug)	*Arion ater* (terrestrial slug)	*Arion fasciatus* (terrestrial slug)	*Arion vulgaris* (terrestrial slug)	*Deroceras reticulatum* (terrestrial slug)	*Geomalacus maculosus* (terrestrial slug)	
			**Method**	Culture independent	Culture based	Culture based + independent	Culture independent	Culture based	Culture based	Culture based + independent	Culture independent	
			**Bacteria isolated from**	Gut and crop	Gut	Gut and crop	Gut	Gut	Faeces	Gut	Faeces	
**Bacteroidetes**	*Bacteriodales*	*Bacteroidaceae*	*Bacteroides*	*B. sp.*			*B. sp.* + *vulgatus*				*B. sp.*	3
	Flavobacteriales	*Flavobacteraceae*	*Chryseobacterium*				*C. gleum*			*C. sp.*	*C. daeguense* + *sp.*	3
	Sphingobacteriales	*Sphingobacteriaceae*	*Mucilaginibacter*	*M. sp.*			*M. paludis*				*M. calamicampi, mallensis, ximonensis* + *sp.*	3
			Sphingobacterium				*S. spiritivorum*			*S. sp.*	*S. pakistanense, spiritivorum* + *sp.*	3
**Firmicutes**	*Clostridiales*	*Clostridiaceae*	*Clostridium*	Clostridiaceae	*C. sp.*		*C. leptum*				*C. saccharogumia*	4
	Lactobacillales	*Carnobacteriaceae*	*Carnobacterium*							*C. sp.*	*C. sp.*	2
		Enterococcaceae	*Enterococcus*		*E. casseliflavus, malodoratus* + *raffinosus*		*E. casseliflavus* + *sp.*				*E. faecium* + *sp.*	3
		Streptococcaceae	*Lactococcus*	*L. sp.*	*L. lactis*		*L. lactis*				*L. sp.*	4
**Beta-Proteobacteria**	*Burkholderiales*	*Comamonadaceae*	*Comamonas*	*C. sp.*			*C. testosteroni*				*C. sp.*	3
		Oxalobacteraceae	*Janthinobacterium*				*J. sp.*			*J. sp.*		2
**Gamma-Proteobacteria**	*Aeromonadales*	*Aeromonadaceae*	*Aeromonas*	*A. sp.*		*A. hydrohila*	*A.hydrophilia*, *salmonicida* + *sp.*	*A. encheleia*, *hydrophilia*, *media*, *popoffii*, *salmonicida* + *sobria*			*A. media* + *sp.*	5
	Enterobacteriales	*Enterobacteriaceae*	*Buttiauxella*		*B. agrestis* + *noackiae*	*B. agrestis*				*B. sp.*	*B. noackiae*	4
			Citrobacter	*C. sp.*	*C. gillenii* + *sp.*	*C. freundii*	*C. koseri, rodentium, youngae* + *sp.*	*C. freundii*	*C. freundii*		*C. freundii* + *sp.*	7
			Enterobacter		*E. amnigenus*	*Enterobacter sp.*	*E. cloacae, cancerogenus* + *sp.*				*E. aerogenes* + *sp.*	4
			Erwinia			*E. amylovora* + *tasmaniensis*	*E. amylovora, bellingiae, pyrifoliae, tasmaniensis* + *sp.*				*E. sp.*	3
			Escherichia				*E. albertii, coli, fergusonii* + *sp.*		*E. coli*		*E.-Shigella*	3
			Ewingella					*E. sp.*			*E. americana*	2
			Klebsiella	*K. sp.*		*K. sp.*			*K. sp.*			3
			Kluyvera		*K. intermedia*	*K. intermedia*	*K. pneumoniae, variicola* + *sp.*	*K. cryocersens*	*K. sp.*			5
			Obesumbacterium		*O. proteus*							1
			Pantoea			*P. sp. 57917*	*P. ananatis, vagans* + *sp.*					2
			Pectobacterium			*P. carotovorum*	*P. carotovorum, wasabiae* + *sp.*					2
			Rahnella				*R. sp.*			*R. sp.*	*R. sp.*	3
			Raoultella		*R. terrigena*				*R. sp.*		*R. sp.*	3
			Salmonella			*S. sp.*	*S. enterica* + *typhimurium*					2
			Serratia			*S. sp.*	*S. odorifera, proteamaculans* + *sp.*			*S. sp.*	*S. plymuthica* + *sp.*	4
	Pseudomonadales	*Moraxellaceae*	*Acinetobacter*	*A. sp.*		*A. beijerinckii*	*A. baumannii, calcoaceticus, haemolyticus, johnsonii, junii, lwoffii, radioresistens* + *sp.*				*A. indicus* + *sp.*	4
		Pseudamonadaceae	*Pseudomonas*	*P. sp.*			*P. aeruginosa, entomophila, fluorescens, mendocina, palustris, putidae, savastanoi, stutzeri, syringae* + *sp.*	*P. tolaasii*		*P. aeruginosa* + *sp.*	*P. lutea* + *sp.*	5
	Xanthomonadales	*Xanthomonadaceae*	*Stenotrophomonas*				*S. maltophilia*	*S. maltophilia*		*S. sp.*	*S. sp.*	4
